# A pilot study examining Toronto-area family physician perspectives on thyroid neoplasm evaluation

**DOI:** 10.1186/s40463-019-0349-4

**Published:** 2019-05-30

**Authors:** Arjun Kundra, David P. Goldstein, Kimberly Wintemute, Sangeet Ghai, Richard W. Tsang, Karuna Gupta, Donatus R. Mutasingwa, Jeff Weissberger, Ella Huszti, Patrick Brown, Huan Jiang, Anna M. Sawka

**Affiliations:** 1 Division of Endocrinology, Department of Medicine, University Health Network and University of Toronto, Toronto, Ontario Canada; 2 Department of Otolaryngology and Head and Neck Surgery, University Health Network and University of Toronto, Toronto, Ontario Canada; 3 Department of Family Medicine, University of Toronto, Toronto, Ontario Canada; 4 Department of Medical Imaging, University of Toronto, Toronto, Ontario Canada; 5 Department of Radiation Oncology, University Health Network and University of Toronto, Toronto, Ontario Canada; 6 Biostatistics Research Unit, University Health Network, Toronto, Ontario Canada; 7 Dalla Lana School of Public Health, University of Toronto, Toronto, Ontario Canada; 8 Cancer Care Ontario, Toronto, Ontario Canada

**Keywords:** Thyroid nodule, Thyroid cancer, Thyroid ultrasound, Fine needle aspiration biopsy

## Abstract

**Objective:**

The incidence of thyroid cancer (TC) is known to be very high in the Greater Toronto Area of Ontario, Canada. We performed a pilot survey study examining Toronto-area family physician (FP) perspectives on thyroid neoplasm evaluation (i.e. thyroid nodules [TNs] or thyroid cancer [TC]) in this region, to explore for potential factors leading to overdiagnosis.

**Methods:**

We performed a cross-sectional mail-out written survey of a random sample of 300 FPs in active practice in the Greater Toronto Area (Markham and Brampton).

**Results:**

The overall response rate was 22.3, 95% confidence interval (CI) 18.0, 27.4% (67/300); the effective response rate was 19.9, 95% CI 15.7, 24.9% (58/291), after excluding 6 FPs that reported TN evaluation was outside their scope of practice and three FPs with an invalid mailing address. There were no missing responses to questions. The demographic characteristics were as follows: 58.6% (34/58) from Markham, 55.2% (32/58) were female, 58.6% (34/58) were in practice > 10 years, and 32.8% (19/58) affiliated with a University. All FPs reported easy access to thyroid ultrasound (TUS). About half of FPs were concerned about overdiagnosis of TC and most did not believe that there was any TC survival advantage with routine screening TUS. Although appropriate indications for TUS were endorsed by most respondents (e.g. palpable TN, incidental TN on other imaging), inappropriate recommendations were observed in a third of FPs (19/57) who recommended TUS for abnormal thyroid blood tests about half of FPs (30/56) who endorsed biopsy of sub-centimeter nodules. About half of FPs (31/58) reported that their patients sometimes request medically unnecessary TUS.

**Conclusion:**

There are likely multiple complex factors leading to potential overdiagnosis of TC in primary care, including some physicians’ knowledge gaps about appropriate indications for TN investigations as well as patients’ requests and expectations.

## Introduction

Thyroid cancer (TC) incidence has been rising in Canada in recent decades, whereas mortality rates have remained relatively low and stable [1]. Topstad and Dickinson suggested this trend may reflect overdiagnosis of low risk lesions, detected by modern diagnostic imaging [1]. Overdiagnosis of neoplasms may lead to overtreatment, without an expected survival benefit. Hall et al. have reported that in Ontario, the highest TC incidence rates were reported a positive relationship between TC incidence rate and regional use of discretionary medical tests (e.g. diagnostic imaging) or thyroid fine needle aspiration biopsies [2]. Among Canadian municipalities, the Greater Toronto Area is known to have the he highest TC incidence [3]. Our objective was to perform a pilot study, surveying a sample of FPs in the Greater Toronto Area about their practices and beliefs relating to evaluation of thyroid neoplasms (including thyroid nodules [TNs] and TC), in order to explore potential factors leading to overdiagnosis of TC in our region.

## Methods

### Setting, sampling frame, and population inclusion/exclusion criteria

The study design was a cross-sectional mail-out, written survey of a sample of FPs in Markham and Brampton, which are municipalities in the Greater Toronto Area. The incidence of TC is relatively higher in Markham and lower in Brampton, relative to the Toronto core region [3]. The Toronto core region was not sampled due to its known high density of thyroid cancer specialty care centres. The study was approved by the University Health Network Research Ethics Board. FPs in active practice in the regions of interest were identified using the 2018 publicly available, register of the College of Physicians and Surgeons of Ontario [4]. The inclusion criteria were FPs in active practice, with a valid postal mailing address in the regions of interest. FPs who reported not seeing any TNs or TC in their scope of practice were excluded. A random sample of 150 FPs/municipality was respectively selected using a random number generator [5] from a total of 274 FPs in Markham and 574 FPs in Brampton.

### Development of the survey instrument

The paper-based, self-administered questionnaire was developed by a multidisciplinary team of all the authors and it was pilot-tested by four family physician authors (KW, KG, DM, and JW), who all reported < 5 min completion time. The first page of the survey included 8 questions on demographic and practice characteristics. The second page included 8 questions requesting opinions on TN evaluation in primary care (agreement scored on a 5-point Likert scale, ranging from 1 = strongly disagree to 5 = strongly agree, as well as a “don’t know” categorical response).

### Survey distribution

In order to improve response rate, we enclosed a cover letter and self-addressed stamped envelope and performed a second mail-out to non-responders [6]. The first mail-out was on May 11, 2018 and the second on June 8, 2018. FPs were not reimbursed.

### Statistical analysis strategy and sample size justification

Data was entered and statistical analyses were conducted in an electronic spreadsheet (Microsoft Excel). We reported all data descriptively (i.e. numbers and percentages (with 95% confidence intervals CI) for the response rate and for categorical data and Likert scale categories, as well as means with standard deviations for Likert scale data). Any “don’t know” responses were reported, but not included in any Likert scale mean calculations or categorical descriptions of opinion statements.

The feasibility criteria for this pilot study proceeding to a larger study included: a response rate of > 45% and ≤ 20% missing responses. For a sample size of 300 and response rate of 52.2%, we estimated 80% power for rejecting values response rate values ≤45% (1-sided type I error rate of 0.05 in a single sample binomial test).

## Results

The overall response rate was 22.3, 95% confidence interval (CI) 18.0, 27.4% (67/300); the effective response rate was 19.9, 95% CI 15.7, 24.9% (58/291), after excluding 6 FPs who did not treat patients with TNs and three FPs with an invalid mailing address. There were no missing responses.

### Demographics and FP practice characteristics

The demographic and practice characteristics of respondents are shown in Table 1. More than half of respondents were in practice longer than a decade and a minority 32.8% (19/58) were affiliated with a University. The most frequently reported predominant practice ethnicity was Asian (39.7%, 23/58). All FPs (100%, 58/58) reported easy access to thyroid ultrasound (TUS), and most reported easy access to TN ultrasound-guided fine needle aspiration biopsy (79.3%, 46/58), Endocrinologists (84.5%, 49/58), and Thyroid Surgeons (58.6% 34/58).

**Table 1:** Characteristics of the Family Physicians (FPs) and their practices

Variable	Percentage (number) (*N* = 58)
Location of practice	Brampton – 41.4% (24)
	Markham – 58.6% (34)
Female gender	55.2% (32)
Years practicing Family Medicine	Less than 5 years – 20.7% (12)
	5 to 10 years - 20.0% (11)
	11 to 19 years – 19.0% (12)
	More than 20 years – 39.7% (23)
University affiliation	32.8% (19)
Most common ethnic background of patients	Caucasian – 22.4% (13)
	Asian – 39.7% (23)
	Hispanic – 0% (0)
	Black – 0% (0)
	Mixed (no predominance) – 36.2% (21)
	Other (“African”) – 1.7% (1)
Number of patients with thyroid nodules or thyroid cancer seen in a typical month	0 to 1–53.4% (31)
	2 to 5–39.7% (23)
	6 to 10–1.7% (1)
	11 to 20–3.4% (2)
	21 to 50–1.7% (1)
	More than 50–0% (0)
Services that are easy to access in the vicinity of FP practice	Thyroid ultrasound – 100% (55)
	Ultrasound-guided fine needle aspiration of the thyroid – 79.3% (46)
	Endocrinology consultation – 84.5% (49)
	Thyroid surgeon – 58.6% (34)
Order or perform biopsies of the thyroid	39.7% (23)
Follow some thyroid cancer survivors in FP practice (without concurrent specialist involvement)	32.8% (19)

### FP opinions on thyroid nodule evaluation in primary care

FPs’ opinions on statements relating to management of TNs are reported in Table 2. FPs expressed the highest degree of uncertainty in deciding whether screening TUS (in asymptomatic individuals) is associated with a reduction in TC deaths or not, with approximately a quarter of respondents (27.6%, 16/58) reporting not knowing the answer. However, the relatively low mean score (standard deviation, SD) for this question (mean 2.4, SD 1.1) suggested that FPs did not believe there was a survival benefit of screening TUS. The majority of respondents agreed or strongly agreed with established indications for TUS, specifically, evaluation of a palpable thyroid nodule (96.6%, 56/58) or investigation of TNs detected incidentally on chest computerized tomography (71.4%, 40/56). Furthermore, the highest mean (SD) score for agreement was expressed for the statement on a palpable nodule indication for TUS – mean 4.7 (SD 0.5). Approximately a third (19/57) of respondents endorsed performing TUS for the inappropriate indication of abnormal thyroid blood tests. More than half of FPs (53.4%, 31/58) reported that some of their patients expect or request a TUS, even if not medically necessary. In terms of TN biopsy indications, the vast majority of FPs (89.7%, 52/58) agreed or strongly agreed that a biopsy should be performed if recommended by a radiologist on an imaging report. However, about half of respondents (53.6%, 30/56) agreed or strongly agreed with biopsy for sub-centimeter suspicious TNs. Moreover, about half of FPs (53.6%, 30/56) agreed or strongly endorsed concern about the possible risk of overdiagnosis of low risk TC.

**Table 2:** Family Physicians opinions on thyroid nodule evaluation in primary care

Statement	Percentage strongly disagree or disagree^a^ (number)	Percentage neutral^a^ (number)	Percentage strongly agree or agree^a^ (number)	Percentage indicating “don’t know”^a^ (number)	Mean (standard deviation)^a^
Thyroid ultrasound (TUS) should be offered for palpable thyroid nodules (TNs)	0% (0/58)	3.5% (2/58)	96.6% (56/58)	0% (0/58)	4.7 (0.5)
TUS should be performed in patients with abnormal thyroid blood tests	40.4% (23/57)	26.3% (15/57)	33.3% (19/57)	1.7% (1/58)	2.9 (1.1)
Some of my patients expect or request a TUS, even if not medically necessary	29.3% (17/58)	17.2% (10/58)	53.4% (31/58)	0% (0/58)	3.3 (1.2)
TC screening by TUS is associated with reduced TC deaths	61.9% (26/42)	16.7% (7/42)	21.4% (9/42)	27.6% (16/58)	2.4 (1.1)
Suspicious TNs < 1 cm should always be biopsied	28.6% (16/56)	17.9% (10/56)	53.6% (30/56)	3.4% (2/58)	3.3 (1.1)
TN should be biopsied, if recommended by a radiologist on an imaging report	0% (0/58)	10.3% (6/58)	89.7% (52/58)	0% (0/58)	4.3 (0.6)
If a TN is incidentally noted on a chest computerized tomography scan, a TUS must be ordered	19.6% (11/56)	8.9% (5/56)	71.4% (40/56)	3.6% (2/58)	3.7 (1.1)
I am concerned about the risk of possible overdiagnosis of low risk TC patients	16.1% (9/56)	30.4% (17/56)	53.6% (30/56)	3.4% (2/58)	3.5 (0.9)

Legend: ^a^Excludes don’t know responses

## Discussion

This exploratory pilot survey study examined opinions of FPs in municipalities in the Greater Toronto area regarding TN evaluation. Our key findings are that FPs in the surveyed regions had easy access to TUS and most had easy access to thyroid specialists (e.g. Endocrinologists, Thyroid Surgeons). About half of FPs expressed concern about potential overdiagnosis of low risk TC. The FPs in our study largely did not believe that screening TUS was associated with a survival benefit and is consistent with recent recommendations by the United States Preventative Task Force [7]. Indications for TUS that are consistent with recent clinical practice guidelines [8], and that were endorsed by most FPs, included evaluation of palpable TNs and incidental TN detection on computerized tomography imaging. However, the endorsement by about half of FPs for the routine biopsy of all TNs < 1 cm in diameter, is inconsistent with recent clinical practice guidelines [8]. In a recent American survey, 48% of primary care physicians were not aware of Endocrine/Thyroid society specialty guidelines on thyroid nodules, and 65% reported not reading them [9]. Approximately a third of respondents in our study endorsed TUS evaluation for patients with abnormal thyroid function studies, which contrasts with a recent Choosing Wisely Canada Endocrinology recommendation [10]. In a prior study from Israel, 30% of patients referred by Primary Care Physicians to Endocrinologists, had a TUS ordered by their primary care doctor because of thyroid dysfunction [11]. In a study from Nova Scotia, Landry et al. reported that the rate of inappropriate TUS ordering by Family Physicians was 18.8%, with some of the reasons including ordering the wrong tests for the clinical query, vague clinical questions, and unnecessary tests for the presenting problem [12]. Some potential strategies for FP-directed knowledge translation on diagnosis and management of TNs, could include: large or small-group learning sessions on this topic at FP conferences or other teaching sessions/rounds, development and dissemination of decision support tools to enable FP communication with patients about appropriate TN management, and engagement of FP leaders/organizations in formulating dissemination and implementation strategies of established TN clinical practice guidelines.

About half of FPs reported that their patients expected or requested TUS, even when not medically necessary. Hendee et al. has reported that patients sometimes demand imaging procedures because they may have heard or read about them (including on the internet) and although physicians have a responsibility to educate their patients, structuring of healthcare reimbursement systems may limit this ability [13]. The local ethnic/cultural context of patients’ attitudes and beliefs on screening and diagnostic imaging is not known.

Strengths of our study include multi-disciplinary team involvement in development of the survey instrument, careful sampling of regions of interest, and no missing responses. The main limitation was the low response rate and sample size. Another limitation was lack of standardized terminology for terms such as easy access. Our findings require confirmation in other regions (such as the Toronto central core area, other parts of the Greater Toronto area, other parts of Canada, and other countries). Furthermore, an in-depth qualitative study of Family Physicians’ opinions may complement such research.

## Conclusions

FPs in the Greater Toronto area are concerned about potential overdiagnosis of low risk PTC. Possible contributing factors to TC overdiagnosis in this region could include some patients requesting unnecessary TUS, some FPs ordering TUS for questionable indications (e.g. abnormal thyroid blood tests), as well as biopsy of sub-centimeter thyroid nodules. Family physician and patient-directed knowledge translation strategies are needed to mitigate potential TN over-investigation that may lead to TC overdiagnosis.

## Data Availability

The datasets generated and/or analysed during the current study are not publicly available per agreement of confidentiality with the research ethics board and participants, but de-identified aggregate data are available from the corresponding author on reasonable request.

## Abbreviations

FPs: Family Physicians
TC: Thyroid cancer
TNs: thyroid nodules
TUS: Thyroid ultrasound
